# 2207. Impact of an Intervention Bundle on Azithromycin Use and 5-Day Durations of Therapy for Ambulatory Pediatric Community Acquired Pneumonia

**DOI:** 10.1093/ofid/ofad500.1829

**Published:** 2023-11-27

**Authors:** Matthew J Weber, Christine MacBrayne, Meghan C Birkholz, Nicole M Poole

**Affiliations:** University of Colorado/Children's Hospital Colorado, Aurora, Colorado; Children's Hospital Colorado, Aurora, Colorado; Children's Hospital Colorado, Aurora, Colorado; University of Colorado School of Medicine, Aurora, Colorado

## Abstract

**Background:**

Amoxicillin for a 5-day duration is the first-line recommended treatment for pediatric uncomplicated community acquired pneumonia (CAP). This project sought to identify the impact of a bundled intervention on first-line treatment for pediatric CAP in ambulatory settings.

**Methods:**

The bundled intervention included (1) an electronic medical record (EMR) order set that preselected a 5-day duration of therapy for patients prescribed an antibiotic for CAP (8/2020) and (2) revision of an existing clinical pathway for CAP (4/2021) that removed amoxicillin-clavulanate and azithromycin as recommended antibiotics.

A retrospective review was conducted between July 2018 and July 2022. Patients ages 60 days to 18 years with an in-person or remote ambulatory encounter and validated ICD10 diagnosis code of pneumonia were included. Hospitalized patients and those with complex chronic conditions were excluded. Data was extracted from EPIC EMR.

Primary outcomes were rates of amoxicillin and azithromycin use (encounters receiving the antibiotic of interest out of all encounters receiving an antibiotic) and 5-day duration prescriptions (encounters receiving a 5-day prescription [excluding azithromycin] out of all encounters receiving an antibiotic). Receipt of an antibiotic by demographic categories were compared by chi-squared test. Statistical process control charts with upper and lower control limits and identified “special cause” variation were used to analyze outcomes.

**Figure:**
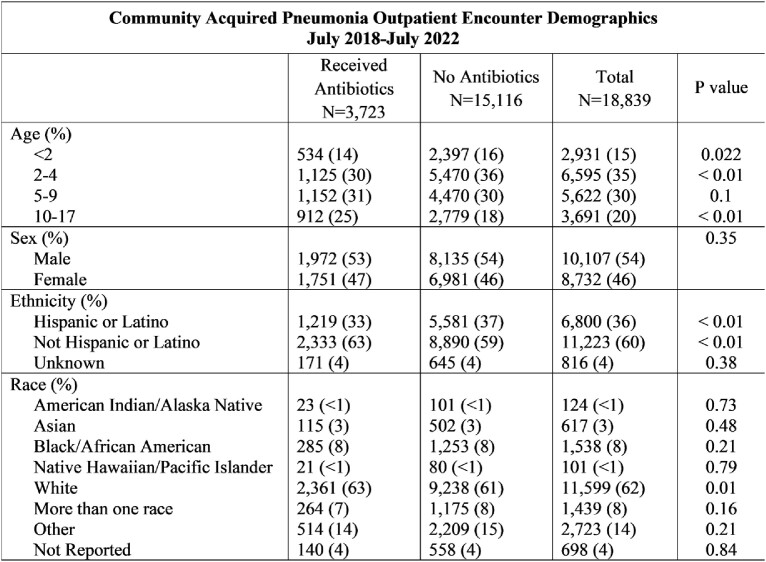

**Results:**

A total 18,839 pneumonia encounters were included with 3,723 (19.7%) prescribed at least one antibiotic. After pathway implementation, the amoxicillin prescribing rate rose from 72 to 80% (p < 0.01) and the azithromycin prescribing rate decreased from 13% to 4% (p = 0.01) (Graphs 1,2). Special cause variation was identified in 5-day prescriptions, which increased from 15% to 58% post-intervention (p < 0.01) (Graph 3).

**Figure:**
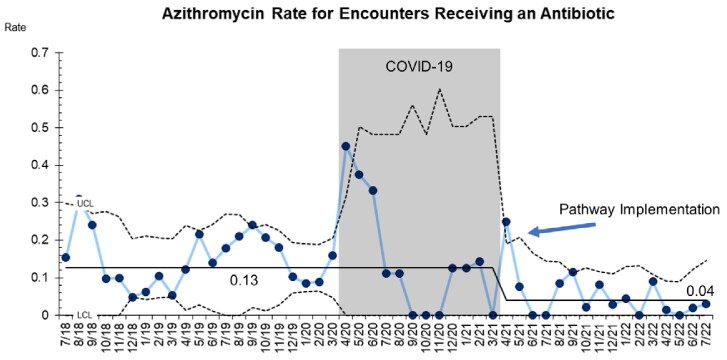

**Figure:**
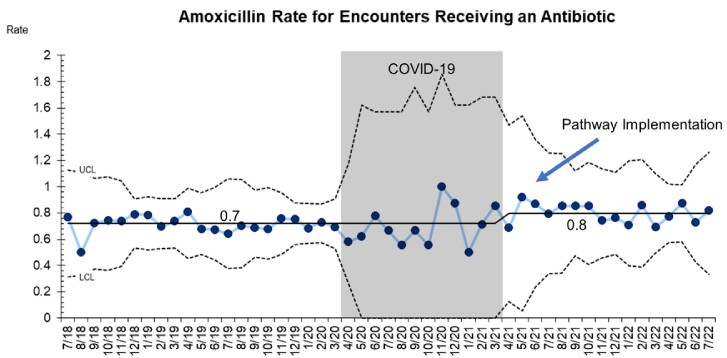

**Figure:**
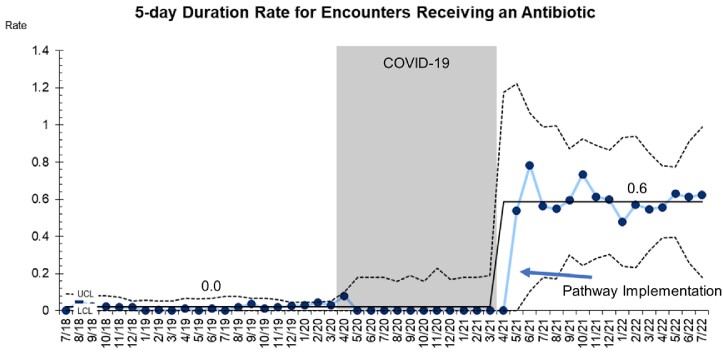

**Conclusion:**

Implementation of a revised clinical pathway substantially increased the rate of 5-day prescriptions for uncomplicated CAP. An EMR order set did not change durations of therapy, likely impacted by the COVID pandemic. Amoxicillin prescribing increased and azithromycin prescribing was negligible after the pathway implementation.

**Disclosures:**

**All Authors**: No reported disclosures

